# Transcriptomic characterization of posterior capsule, anterior capsule, and femoral condylar tissue highlights tissue-specific microenvironments in knee osteoarthritis

**DOI:** 10.1016/j.gendis.2025.101530

**Published:** 2025-01-10

**Authors:** Odette Laneuville, Daniel Stratis, Guy Trudel, Robert Feibel, T Mark Campbell

**Affiliations:** a Department of Biology, University of Ottawa, Ottawa, ON K1N 6N5, Canada; b Bone and Joint Research Laboratory, Ottawa Hospital Research Institute, Department of Cellular and Molecular Medicine, Ottawa, ON K1H 8L1, Canada; c Faculty of Medicine, University of Ottawa, Department of Medicine, Division of Physical Medicine and Rehabilitation, Ottawa, ON K1H 8M5, Canada; d The Ottawa Hospital, Department of Medicine, Division of Physical Medicine and Rehabilitation, Ottawa, ON K1H 8M2, Canada; e Department of Physical Medicine and Rehabilitation, Elisabeth Bruyère Hospital, Ottawa, ON K1N 5C8, Canada

Knee osteoarthritis (OA) is extremely common and often complicated by loss of extension (flexion contracture or FC), associated with worse clinical outcomes.^1^, ^2^, ^3^ Once established, an FC is very difficult to reverse, leading to increased long-term morbidity.^1^, ^2^, ^3^ Whole-genome microarray studying posterior knee capsule biopsies in people with OA and knee FC reported an up-regulation in cellular and biological adhesion pathways consistent with an underlying activated connective tissue process, such as excess fibrous tissue production, limiting knee extension.^3^ Fibrous tissue is produced by fibroblasts, which in turn are derived from mesenchymal stromal cell precursors.^4^ Fibroblasts and mesenchymal stromal cells may therefore contribute to a pathologic tissue process in the posterior joint capsule in OA, such as capsular fibrosis, and could represent novel early targets for reducing OA morbidity. Therefore, the objectives of this study were to examine the whole-genome expression of fibroblasts and mesenchymal stromal cells within the posterior and anterior OA joint capsule and to identify genes and pathways associated with knee FC to better understand the pathophysiology of OA FC development and direct novel treatment avenues.

Whole genome data was obtained from the Ottawa Knee OA (OKOA) database, a primary OA tissue bank and database evaluating clinical and laboratory biomarkers of knee OA and range of motion. For the present study, we included 12 participants who provided samples from 3 different tissues during total knee arthroplasty: anterior and posterior knee capsule, as well as distal femoral bone fragment (3 tissue samples per participant, giving 36 tissue samples total). All participants met the American College of Rheumatology criteria for knee OA.^5^ Participant demographics are summarized in Table S1. Single-end sequencing of fibroblasts and mesenchymal stromal cells was performed on the NextSeq 500 System platform using a NextSeq 500/550 High Output v2 75 cycles sequencing kit. Sequencing results for the 36 samples after filtering provided 14,612 protein-coding genes. Mesenchymal stromal cell identity was confirmed using trilineage differentiation capacity and flow cytometry. The vast majority of cells expressed osteocalcin and aggrecan following differentiation, while roughly half expressed fatty acid binding protein 4 (FABP4) (Fig. S1). For flow cytometry, approximately 40% of plastic-adherent cells from each tissue met the International Society for Cell & Gene Therapy definition for mesenchymal stromal cells^5^ with variability across individual participants (posterior capsule 36.6% ± 23.8%, anterior capsule 38.7% ± 30.7%, and bone 40.8% ± 29%). Principal component analysis revealed clustering by sample site with 38.3% and 13% of the variance explained by principal components 1 and 2, respectively (Fig. 1A). The 95% confidence intervals of all samples overlapped significantly; all but two samples from the anterior capsules were included in the regions of the bone and posterior capsule ellipses (Fig. 1A). The principal component analysis plot was contrasted with the sample t-SNE (t-distributed stochastic neighbor embedding) map (Fig. 1B). Non-linear clustering of samples (t-SNE) grouped by tissues showed a better separation compared with linear principal component analysis. Comparing the transcriptomes of bone, anterior capsule, and posterior capsule identified 3456 differentially expressed protein-coding genes (Fig. 1C and Table S2). There were more transcriptome differences between bone and capsule: anterior capsule versus bone had 985 differentially-expressed genes, and posterior capsule versus bone had 2554 genes; while posterior versus anterior capsule showed only 52 differentially-expressed genes (Fig. S2 and Table S2). Eleven genes were differentially expressed between participants with and without FC, across tissue types (Fig. 1D). The effects of knee FC on the transcriptomes of the posterior capsule identified up-regulated expression of KCNK2, ACTN2, SOX17, and CCDC144A genes, and down-regulated expression of HES2, GSTM1, MAB2L3, ZNF497, and SRXN1 genes in the FC group compared with no FC; there was an opposite pattern for these genes in the anterior capsule (Fig. 1D and Table S2). When tissue type was removed from the analysis, five genes were differentially expressed between participants with and without FC: up-regulation of DDX3Y and GSTM1 genes and down-regulation of KDR, ACTN2, and MAB21L3 genes in the FC group (Fig. 1D and Table S2). It was surprising that knee FC was associated with only a handful of gene expression alterations. The down-regulation of kinase insert domain receptor (KDR), a tyrosine-protein kinase that acts as a cell-surface receptor for VEGFA, VEGFC, and VEGFD and plays an essential role in the regulation of angiogenesis, may suggest that pathologic angiogenesis in OA may be positively or negatively influenced by knee biomechanics.

**Figure 1:**
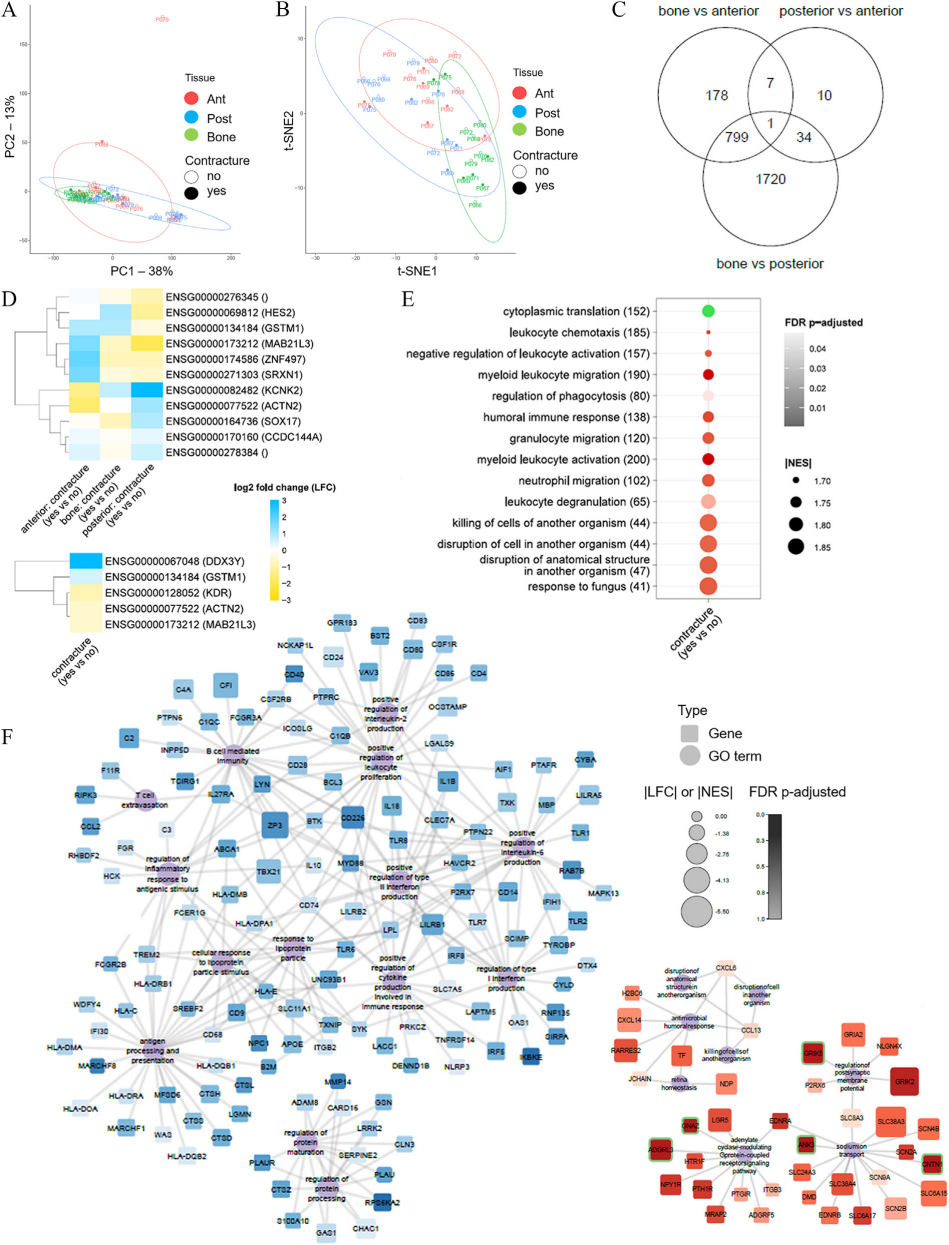
Differential gene expression and enrichment analysis of transcriptomes from anterior and posterior knee capsule tissues. **(A)** Linear principal component analysis (PCA) and (B) bon-linear t-distributed stochastic neighbor embedding (t-SNE) were used to visualize cluster classification across all samples. Datasets correspond to the normalized read counts for the 14,612 protein-coding gene list and the 95% confidence is displayed as ellipses. Each point represents a participant sample from either bone (green), posterior capsule (blue), or anterior capsule (red). Samples from participants with or without flexion contracture (FC) are indicated with closed circles or open circles, respectively. **(C)** The Venn diagram showing the number of differentially- and commonly-expressed genes identified from pairwise comparisons. **(D)** Differentially expressed genes were identified using a generalized linear mixed model (GLMM): read counts ∼ tissue + FC + tissue:contracture + (1 | patient) + ϵ. The heatmaps display log-fold change (LFC) values for differentially expressed genes of the anterior capsule, bone, and posterior capsule tissues (upper panel) and for participants with or without FC (lower panel). Human Genome Organisation Nomenclature Committee (HGNC) and gene symbols are indicated in parentheses. Blue indicates gene up-regulation in the posterior versus anterior capsule, and yellow indicates gene down-regulation. **(E)** Functional terms identified through the gene set enrichment analysis (GSEA) of the 14,612 protein-coding genes between FC versus No FC group for the 3 tissues (left panel) and between bone versus anterior capsule and bone versus posterior capsule (right panel). Redundant gene ontology (GO) terms were removed using Lin's semantic similarity measure. The number of genes associated with each GO term is indicated in parenthesis. Absolute value normalized enrichment scores (|NES|) are proportional to the size scale and color (green for positively enriched GO terms and red for negatively enriched GO terms). False discovery rate (FDR) adjusted *P*-values are indicated according to the gradient scale where darker points have lower values. **(F)** Positively (upper left) and negatively (lower right) enriched GO terms from the GSEA of 14,612 protein-coding gene LFC comparing posterior versus anterior tissue capsules. Networks were constructed using the enriched GO terms from GSEA with redundant terms removed using Lin's semantic similarity measure. Functional terms are represented as circles and genes as squares. Shape sizes are proportional to the |NES| for functional terms |LFC| for genes, respectively. Color gradient represents the FDR adjusted *P*-values, where darker points have lower values (color scale). Green frames around squares indicate differentially expressed genes based on the criteria of |LFC| >0.5 and FDR corrected *P*-value <0.1.

We next examined the profiles of 14,612 genes, differentially expressed or not, which were used for gene set enrichment analysis (GSEA) using clusterProfiler's gseGO() function in the R environment with default parameters of 1000 permutations, maximum term size of 500, and a minimum term size of 10. Differentially expressed genes from the 3 tissues identified a single positively enriched gene ontology (GO) term that differentiated between the FC group and no FC group: “cytoplasmic translation” (Fig. 1E). The remainder of the 13 GO terms were negatively enriched, all of which were related to immune processes (Fig. 1E). Functional analysis of the differentially expressed genes identified by comparing bone versus the two capsule tissues identified GO terms with the highest positive score (reflecting up-regulation in bone versus capsule) included “cartilage development”, “appendage morphogenesis”, and “bone growth” (Fig. S3). GO terms with negative scores (reflecting up-regulation in capsule versus bone) were related to the immune processes including “myeloid leukocyte activation”, “regulation of leukocyte mediated immunity”, and “interleukin-6 production” (Fig. S3).

Regardless of gene differential expression and statistical significance, gene sets were created by mapping the 14,612 genes to their associated GO terms across any level under “biological processes”. Perhaps the most striking and unexpected finding in this study was that rather than identifying activated fibrotic pathways, we found marked differences in immune-related pathways. GO terms with positive normalized enrichment scores included immune-related processes such as the production of cytokine, interleukin-6, and interleukin-2 as well as B and T cell-mediated immunity functions (Fig. 1F, left). GO terms with negative normalized enrichment scores demonstrated two functional categories including function related to immune regulation, membrane transport, cell signaling, and sodium transport (Fig. 1F, right).

In summary, we identified only 11 differentially expressed genes in the posterior capsule between participants with and without FC, suggesting that FC development may be multifactorial and involve alterations in articular tissues in addition to the capsule (*e.g.*, osteophyte formation, synovial inflammation, and joint effusion). While the anterior capsule also demonstrated enrichment in immune-modulating GO terms versus bone, enrichment analysis of transcriptomes from posterior and anterior capsule tissues demonstrated a greater number of positively enriched GO terms associated with enhanced immune response in the posterior capsule. Advancing OA is associated with increased synovial proliferation, fibrosis, and infiltration with immune cells such as macrophages and lymphocytes. Our study highlights that OA is a total joint disease and suggests that each articular tissue may contribute uniquely to OA symptoms and progression, raising the possibility that tissue-specific treatments may be necessary to control the disease.

## Ethics declaration

The OKOA was approved by the local institutional research ethics board (protocol 20140139-01H). All participants provided written consent for tissue collection and gene expression analysis.

## Funding

This study is funded by the Bruyère Academic Medical Organization Research Innovation Fund (Ottawa, Canada) (No. BAM-18-001). The funding source had no role in the design of this study, analysis of the data, or production of the manuscript.

## CRediT authorship contribution statement

**Odette Laneuville:** Writing – review & editing, Software, Resources, Methodology, Investigation, Formal analysis. **Daniel Stratis:** Methodology, Investigation, Formal analysis. **Guy Trudel:** Writing – review & editing, Methodology, Investigation. **Robert Feibel:** Writing – review & editing, Methodology, Investigation. **T Mark Campbell:** Writing – review & editing, Writing – original draft, Visualization, Validation, Supervision, Software, Resources, Project administration, Methodology, Investigation, Funding acquisition, Formal analysis, Data curation, Conceptualization.

## Conflict of interests

The authors have no competing interests to disclosed.
