# Supplementary material for: Transcriptomic characterization of posterior capsule, anterior capsule, and femoral condylar tissue highlights tissue-specific microenvironments in knee osteoarthritis
Source: Genes Dis. 2025 Jan 10;12(5):101530. doi: 10.1016/j.gendis.2025.101530 (PMC12167082; doi:10.1016/j.gendis.2025.101530)
Supplement: Multimedia component 1 [file mmc1.docx]

Supplementary Materials

# Materials and methods

*Participant selection and clinical evaluation*

The OKOA is a primary OA tissue bank and database evaluating clinical and laboratory biomarkers of knee OA and ROM. Participants scheduled for arthroplasty were first screened for clinical trial NCT02861521, a trial evaluating post-arthroplasty LLI correction in people with bilateral pre-operative knee FC, and included in the OKOA, if FC was present in only one knee or absent in either. Exclusion criteria comprised history of inflammatory arthritis, previous knee surgery, metastatic cancer or disorders affecting bone. Participants in the OKOA were recruited consecutively from the outpatient orthopedic clinic at The Ottawa Hospital between August 2016 and March 2021. For the present study, we included twelve participants who provided samples from 3 different tissues during total knee arthroplasty: anterior and posterior knee capsule, as well as distal femoral bone fragment (3 tissue samples per participant, giving 36 tissue samples total). The OKOA were approved by the local institutional research ethics board (protocol 20140139-01H). All participants provided written consent for tissue collection and gene expression analysis. All subjects met the American College of Rheumatology criteria for knee OA^28^.

Maximum knee extension was measured using a standardized protocol^8,29,30^. The participant was placed in the supine position with the ankle on a foam roller and participants’ knees were extended as fully as possible. A goniometer was used to measure the knee extension angle with the fulcrum over the knee joint line, the upper arm directed towards the greater trochanter and the lower arm directed towards the lateral malleolus. This method has been shown to have high inter-rater reliability.^31,32^ The inability to extend the knee to 5° constituted a knee FC.^6,31^

*Tissue collection, digestion and tissue culture*

Open posterior knee capsule tissue biopsies approximately 150-200mm^3^ were obtained from the capsule just posterior to the mid-posterior aspect of the medial femoral condyle under direct visualization. Tissue size was chosen based on the maximum size that could be obtained without risking injury to the posterior neurovascular bundle. Anterior capsule was similarly harvested during the initial medial parapatellar arthrotomy incision. Bone samples were obtained from the routine distal femoral bone cuts for component fitting. Samples were collected per-operatively and placed in low-glucose Dulbecco′s Modified Eagle′s Medium (DMEM Gibco, Paisley, UK) for further laboratory processing.

For cellular extraction, tissues were digested in collagenase as described previously^33^. Briefly, capsule and bone samples were weighed, manually divided, then submerged in a minimum volume of StemMACS MSC expansion media (Miltenyi Biotec, Bisley, UK) containing 300U/mL of collagenase (Sigma-Aldrich C0130-100MG) for digest. Tissues were digested at 37°C for 4 hours with interval manual swirling of contents. Following digest, the tissue was plated directly into 6-well tissue culture plates containing StemMACS MSC expansion media. Cells from the supernatant were then plated at 4000-6000 cells per cm^2^ in an appropriately-sized flask, separate from the already-plated post-digest tissue. Post-digest tissue and cell flasks were left for culture expansion for 3 days to minimize the time in tissue culture, while allowing cells within the plated tissue to migrate out and adhere to plastic. Following the 3 days, flasks were washed with PBS to remove any remaining tissue and non-adherent cells. The cells from the tissue + supernatant digest flasks were combined for either flow cytometry and RNA-Seq analyses.

*Cell characterization, differentiation, flow cytometry*

Plastic-adherent cells were characterized using quadrilineage differentiation and flow cytometry. In order to maximize the cells and RNA available for RNA-Seq from the tissue samples, differentiation (n=12; n=6 each for the FC and no FC groups) and flow cytometry (n=28; n=15 for FC group and n=13 no FC group) were performed on samples from different OKOA participants than those whose tissues contributed to RNA-Seq.

For quadrilineage differentiation, passage 3 MSCs were induced towards osteogenesis, chondrogenesis and adipogenesis using standard protocols^34^ and towards fibrogenesis, as described previously^35^, with the cells allowed to adhere to a circular cover slide placed in the respective culture wells. Fibrogenic cultures were grown in DMEM with 10% FBS, antibotics, L-ascorbic acid and recombinant Human CTGF (ThermoFisher Scientific, Ottawa, Canada, PHG0286). For osteogenesis and chondrogenesis, we used StemMACS OsteoDiff and ChondroDiff media, respectively (Miltenyi Biotec); adipogenic cultures were grown in DMEM with 10% FCS, antibiotics, 10% horse serum (Stem Cell Technologies, Vancouver, Canada), 0.5 mM isobutylmethylxanthine, 60 μM indomethacin, and 0.5 μM hydrocortisone (all from Sigma). Assays were performed in duplicate for each sample. Differentiated cultures were visualized using immunofluorescence (IF). Fibrogenic cultures were evaluated using fibroblast-specific antibody (FSP-1) on day 28. Osteoblast differentiation was evaluated labelling with anti-hOsteocalcin antibody (part# 967801, R&D Systems, Minneapolis, MN) day 21 using the standardized protocol^36^. Chondrogenic pellets were cut into five-µm sections using the Leica CM1950 cryostat (Leica Biosystems, Nussloch, Germany), fixed and labelled with anti-hAggrecan (part# 967800, R&D Systems). Adipogenic cultures were labelled on day 21 post-induction with anti-mFABP4 (part #967799) as previously described^36^. Primary antibodies were visualized using NL557-conjugated donkey anti-mouse secondary antibody (Catalog # NL007, R&D systems).

For flow cytometry, cells were allowed to expand to passage 1 to allow for sufficient numbers for analysis. Following tissue culture expansion to passage 1, cells were trypsinized, resuspended in PBS and centrifuged for 5 minutes at 400xg. The resulting pellet was resuspended in FACS buffer, and incubated in a 10% Fc receptor-blocking reagent solution (Miltenyi Biotech) before fluorophore-conjugated antibodies were added, as described previously^33^. Staining was performed for 7AAD, CD90 (Thy1-FITC, Serotec/Bio-rad), CD73 (5′ Ecto-nucleotidase -PE, BD Biosciences), CD45 (PE-Cy7, BD Biosciences), and CD31 (V450, BD Biosciences). The proportion of plastic-adherent cells that met the International Society for Cellular Therapy (ISCT) minimal criteria for defining MSCs was defined as 7AAD^-^CD73^+^CD90^+^CD45^-^CD31^-37^. We attempted to separate cells meeting the ISCT definition for MSC-specific gene expression using FACS, but cell viability was quite low, resulting in significant loss of RNA material. We therefore proceeded with RNA analysis from all plastic-adherent cells. All flow cytometry data were analysed using FloJo software (version 10.2, BD Biosciences).

*RNA isolation, library preparation, and sequencing*

Total RNA was extracted from plastic-adherent cells using TRIzol reagent (Thermo Scientific, Waltham, MA, USA), and genomic DNA was removed by DNase I digestion. Non-RNA contaminants were removed using the Qiagen RNeasy mini kit column (Qiagen Hilden, Germany). RNA was suspended in RNAse-free water and RNA quantity and quality determined using the Agilent Fragment Analyzer (Agilent Technologies, Germany). Not all samples had an RNA Quality Number (RQN) above 8 (mean RQN 4.3±2.0), and some samples did not meet the required 10 ng input threshold. As a result, we opted for ribosomal depletion over poly-A tail selection to prevent the loss of transcripts with lower integrity. Subsequently, up to 10 ng of total RNA and the NEB Ultra II directional RNA kit used for library preparation. Single-end sequencing was performed on the NextSeq 500 System platform (Illumina, San Diego, USA) using a NextSeq 500/550 High Output v2 75 cycles (Illumina, San Diego, USA) sequencing kit according to the manufacturer’s instructions. Library preparation, quality assessment, and high-throughput sequencing were performed at StemCore Laboratories Genomics Core Facility (OHRI, uOttawa), RRID:SCR_012601.

*RNA-sequencing data processing, exploratory analysis, and differential expression analysis*

Gene-level mapping of reads was aligned to the publicly available human genome (GRCh38.84) using HISAT2 with default parameters (v2.2.1) for unpaired reverse complemented reads^38^. Transcript abundance calculations were performed using *featureCounts* default parameters (version 2.0.6)^39^. Gene read counts were normalized for sample read depth using *DESeq2’s* median of ratios. Genes with low read count and insufficient data were excluded. This included genes with average normalized read counts <5 across all samples. This process resulted in a profile of 14,612 genes representing the expressed transcriptome across all samples.

The initial RNA sequence analysis was a linear-based principal component analysis (PCA) which calculates the first principal component with the largest variance and then seeks the second component in the same manner. Additionally, we applied a second method of dimensionality reduction using the non-linear based t-distributed stochastic neighbor embedding (t-SNE) to visualize clusters of samples. Both approaches used the profiles of 14,612 genes for dimensionality reduction.

To identify differentially expressed genes between samples, we applied mixed-effect generalized linear modelling (GLMM) for each of the 14,612 genes using the glmmSeq package in the R environment (Lewis, Myles, Katriona Goldmann, and Elisabetta Sciacca. 2022. glmmSeq: General Linear Mixed Models for Gene-Level Differential Expression. https://CRAN.R-project.org/package=glmmSeq). The normalized read counts from each individual gene were modelled with a negative binomial distribution and fit with both fixed and random effect variables:

Read counts ~ tissue + contracture + tissue:contracture + (1 | participant) + ɛ

Modelling participants as a random-effect allowed us to account for the autocorrelation occurring from participant repeated measures. Adjustment for multiple statistical tests was done using the Benjamini–Hochberg correction^40^. Genes with an absolute value log2 fold change (LFC) >0.5 and false discovery rate (FDR) adjusted P values <0.1 were considered statistically significant and differentially expressed. All sequence analyses and result visualizations were conducted using the R environment (v4.3.1) (https://cran.r-project.org/doc/manuals/r-release/R-intro.html) with custom scripts.

*Enrichment analysis and network visualization*

The profiles of 14,612 genes, whether differentially expressed or not, were used for Gene Set Enrichment Analysis (GSEA) analysis ()^41^. GSEA was carried out with *clusterProfiler’s* gseGO() function in the R environment with default parameters of 1,000 permutations, maximum term size of 500 and a minimum term size of 10^42^. Regardless of gene differential expression and statistical significance, gene sets were created by mapping the 14,612 genes to their associated Gene Ontology (GO) terms across any level under “Biological Processes”. For each gene set, the log2 fold changes (LFCs) between groups were used to rank the genes. The enrichment score (ES) for each gene set was calculated using the entire ranked list and normalized for differences in gene set sizes to determine normalized ES (NES). NES statistical significance was determined through the Kolmogorov-Smirnov (KS) test to determine non-random distribution across each ranked gene set. Gene sets with a FDR adjusted p-value <0.05 were considered statistically significant. Significant GO terms were summarized by cluster networks based on Lin’s semantic similarity measure using REVIGO via a web browser^43^. Further processing of networks for clarity and aesthetics were done using Cytoscape^44^ to illustrate the links between GO terms (circles) and differentially expressed genes (squares).

# Tables

**Table S1. Participant demographics**

| **Demographic feature** | **All**  **mean±SD**  **(n=12)** | **Contracture mean±SD**  **(n=6)** | **No Contracture**  **mean±SD**  **(n=6)** |
| --- | --- | --- | --- |
| Age (years) | 68.6±11.3 | 71.0±9.9 | 66.2±12.9 |
| BMI (kg/m^2^) | 35.2±8.3 | 33.8±8.7 | 36.6±8.4 |
| % female | 42(5/12) | 17(1/6) | 67(4/6) |
| Maximum knee extension (°) | 6.1±6.5 | 11.7±4.1^***^ | 0.5±1.2 |
| Maximum knee flexion (°) | 108.6±14.0 | 101.7±13.7 | 117.0±9.7 |
| KL grade | 3.6±0.5 | 3.7±0.5 | 3.5±0.6 |
| VAS Pain (avg) | 5.4±2.4 | 3.8±1.3^*^ | 7.0±2.1 |
| KOOS Score |  |  |  |
| Pain | 47.5±19.8 | 56.8±15.1 | 36.4±20.3 |
| Symptoms | 45.3±27.3 | 58.5±8.6 | 29.4±34.6 |
| Function | 48.6±26.6 | 60.2±15.3 | 34.8±32.3 |

* p<0.05 contracture versus no contracture group

*** p<0.001 contracture versus no contracture group

BMI: body mass index, KL: Kellgren and Lawrence radiographic osteoarthritis severity grade, KOOS: Knee Injury and Osteoarthritis Outcome Score, SD: standard deviation, VAS: visual analogue pain scale.

**Table S2: Differential expression analysis testing 14,612 protein-coding genes**

| **Variable** | **Comparison** | **Cutoff** | **Gene count** | | |
| --- | --- | --- | --- | --- | --- |
|  |  |  | Up-regulated | Down-regulated | Total |
| Tissue | - | FDR<0.1 | - | - | 3,456 |
|  | Anterior vs bone | FDR<0.1 & \|LFC\| >0.5 | 749 | 286 | 985 |
|  | Posterior vs bone | FDR<0.1 & \|LFC\| >0.5 | 1,619 | 935 | 2,554 |
|  | Posterior vs anterior | FDR<0.1 & \|LFC\| >0.5 | 6 | 46 | 52 |
|  |  |  |  |  |  |
| Contracture | Yes vs no | FDR<0.1 & \|LFC\| >0.5 | 2 | 3 | 5**^α^** |
| Tissue: contracture | Yes vs no | FDR<0.1 | - | - | 11 |

RNAseq was performed on the Illumina NextSeq500 system

Model: read counts ~ tissue + contracture + tissue:contracture + (1 | patient) + ɛ.

FDR: False discovery rate, LFC: Log2 fold change.

**^α^**Differentially expressed genes between contracture and no contracture groups:

**DDX3Y** (DEAD-box helicase 3 Y-linked) - involved in ATP binding, hydrolysis, RNA binding, and in the formation of intramolecular interactions (upregulated in contracture)

**GSTM1** (glutathione S-transferase mu 1) – detoxification function via conjugation with glutathione (upregulated in contracture)

**KDR** (kinase insert domain receptor, VEGFR, VEGFR2) – main mediator of VEGF-induced endothelial proliferation (downregulated in contracture).

**ACTN2** (actinin alpha 2) - actin-binding protein to help anchor actin filaments (downregulated in contracture).

**MAB21L3** (mab-21 like 3) – function not described (downregulated in contracture).

# Figures

**
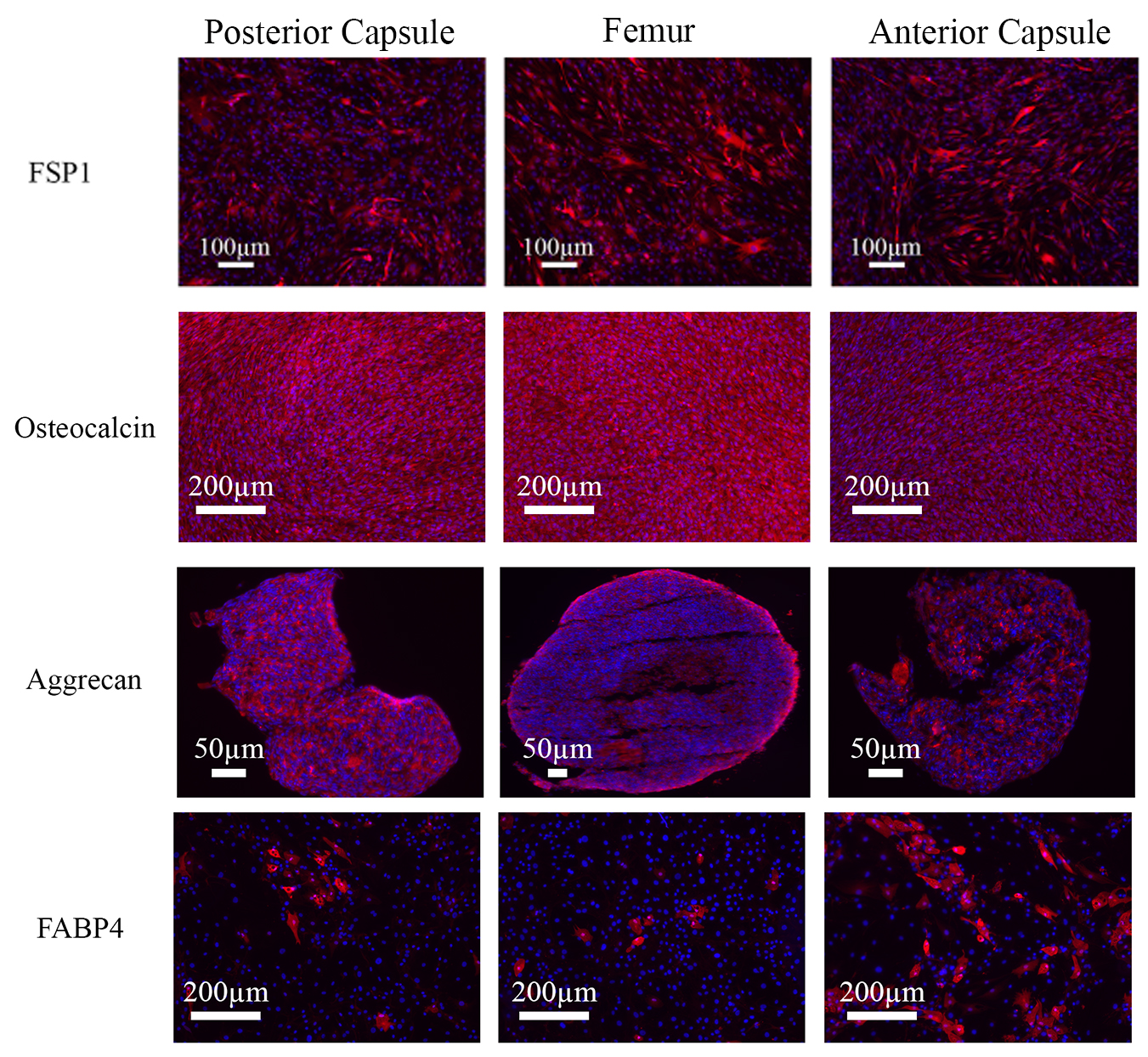
Figure S1**

**Figure S1. Differentiation assays.** Representative immunofluorescence images showing differentiation of MSCs from posterior capsule, distal femoral bone fragment and anterior capsule tissues. Fibroblast differentiation is evaluated using anti-fibroblast specific protein 1 (FSP1) antibody, osteoblast using anti-osteocalcin antibody, cartilage using anti-aggrecan antibody, and adipose using anti-fatty acid binding protein 4 (FABP4).

**Figure S2**


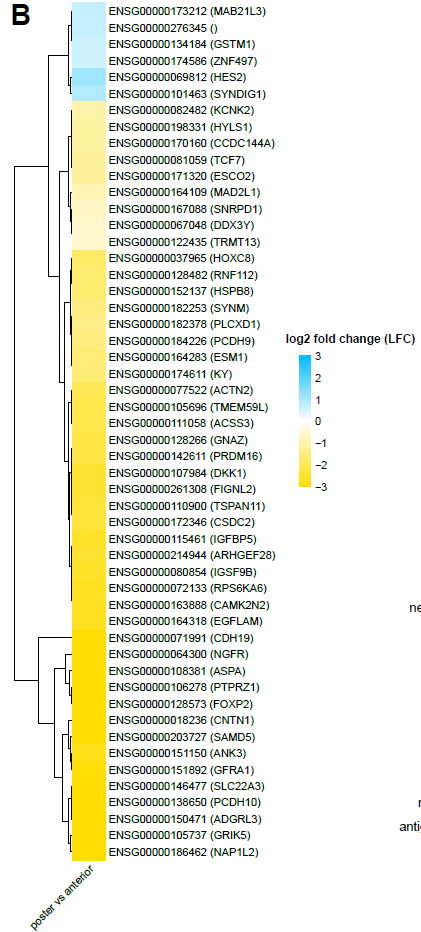

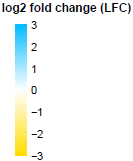


**Figure S2. Tissue transcriptomes.** Differentially-expressed genes identified from posterior and anterior capsule. Differentially-expressed protein-encoding genes (n=52) and their log2 fold changes are displayed in the heatmap for the posterior vs anterior capsule comparison. Human Genome Organisation Nomenclature Committee (HGNC) and gene symbols are indicated in parentheses. Blue indicates gene up-regulation in the posterior vs anterior capsule, yellow indicates downregulation.

**Figure S3**


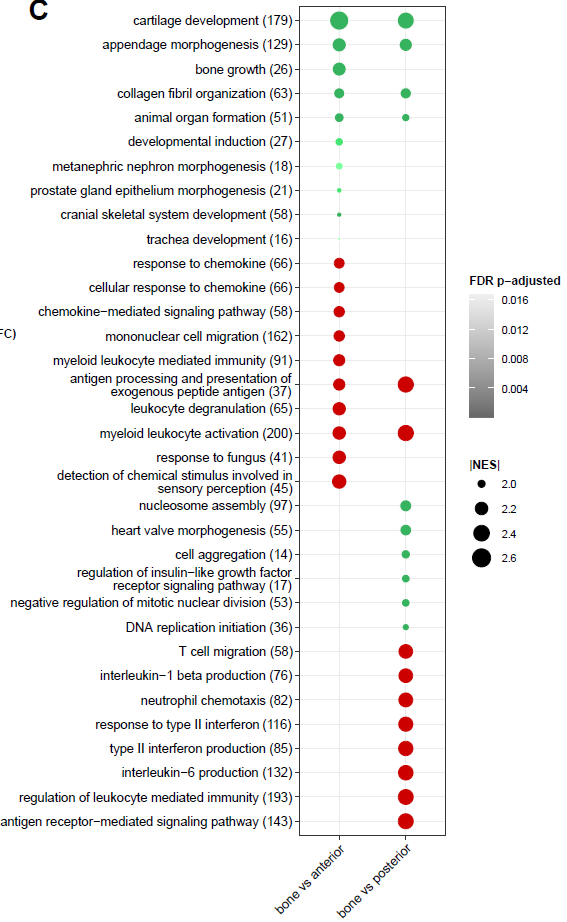


**Figure S3. Differential tissue Gene Ontology**. Enriched Gene Ontology (GO) terms identified through the Gene Set Enrichment Analysis of the 14,612 protein-coding gene log2 fold changes between bone vs anterior capsule and bone vs posterior capsule (green for positively enriched and red for negatively enriched GO terms, respectively). Redundant GO terms were removed using Lin’s semantic similarity measure. The number of genes associated with each GO term indicated in parenthesis. Absolute value normalized enrichment scores (|NES|) are proportional to the size scale and color (green for positively enriched GO terms and red for negatively enriched GO terms). FDR adjusted p-values are indicated according to the gradient scale where darker points have lower values.

FDR: false discovery rate, GSEA: Gene Set Enrichment Analysis, GO: Gene Ontology, HGNC: Nomenclature Committee, Human Genome Organisation: HUGO, NES: normalized enrichment scores.
